# Supplementary material for: MicroRNA Profiles in Intestinal Epithelial Cells in a Mouse Model of Sepsis
Source: Cells. 2023 Feb 24;12(5):726. doi: 10.3390/cells12050726 (PMC10001189; doi:10.3390/cells12050726)
Supplement: Supplementary file 1 [file cells-12-00726-s001.zip › cells-2198459-supplementary.pdf]

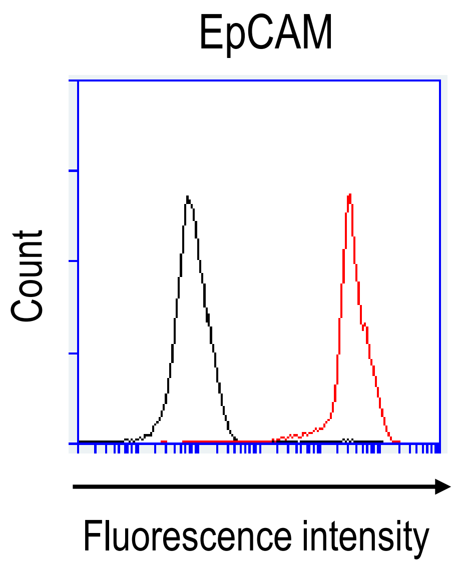

Figure S1. Confirmation of EpCAM expression in the IECs isolated and further enriched with EpCAM microbeads using flow cytometry. The representative histogram is shown. Black line, isotype control; and red line, anti-EpCAM monoclonal antibody.

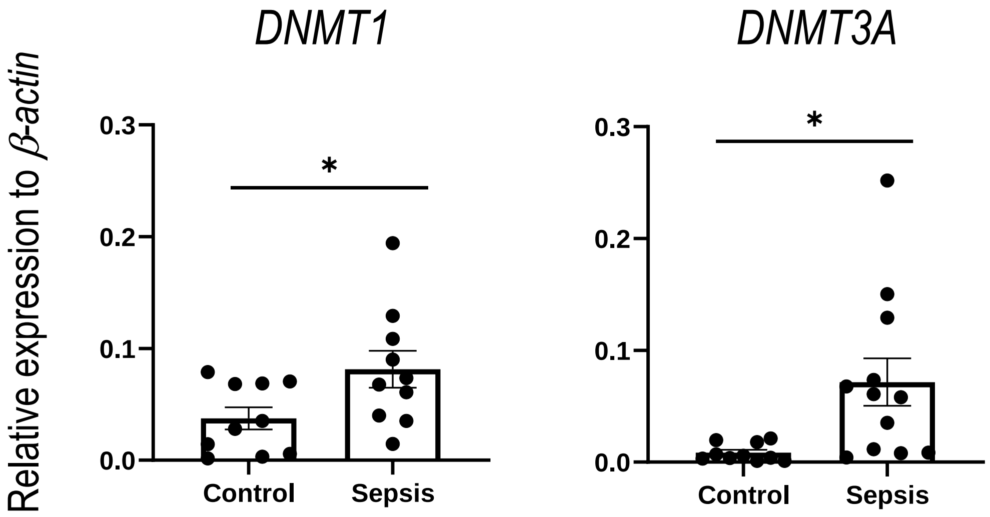

Figure S2. Validation of *DNMT1* and *DNMT3A* expressions. *DNMT1* and *DNMT3A* upregulated in sepsis IECs were confirmed by using real-time quantitative PCR (RT-qPCR) analysis. *b-actin* was used as an endogenous control to normalize mRNA expression levels. Data are shown as scatter plots and bars overlaid with the mean ± standard error of the mean (SEM). Dot on the plot represents the value of each mouse. Control and sepsis indicate the IECs isolated from sham and sepsis cohorts, respectively. *$P < 0.05$.

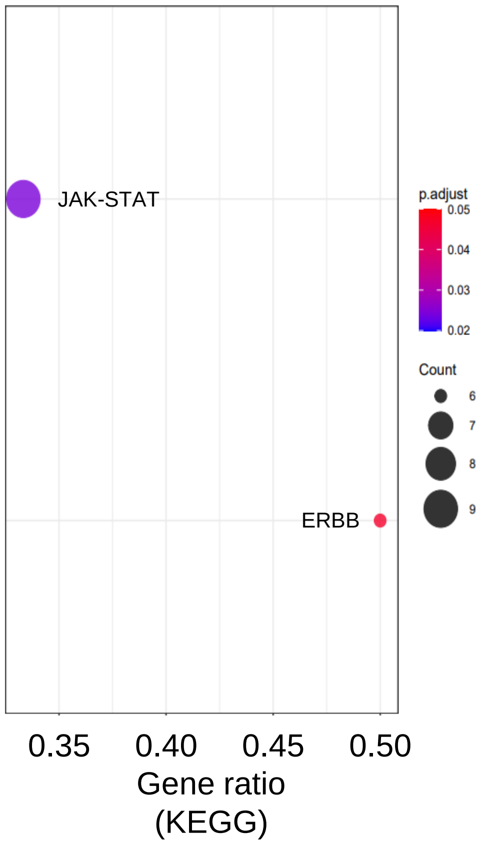

Figure S3. Bioinformatic analysis of downstream signaling and regulatory pathways by downregulated miRNAs with sepsis. KEGG pathway analysis is shown in three-way bubble plot analysis in the basis of gene ratio, gene count, and adjusted p-value.

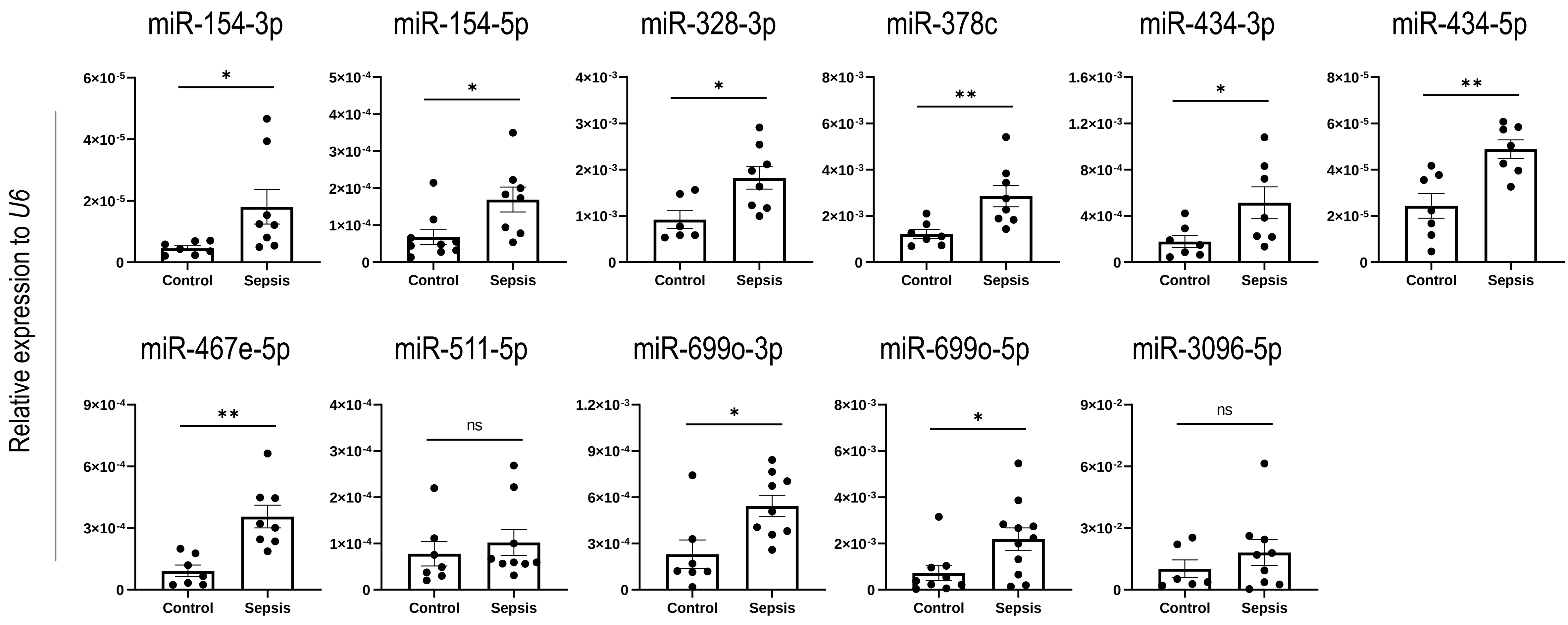

Figure S4. Validation of expression of other sepsis-increased miRNAs. A majority of other miRNAs upregulated in sepsis IECs were confirmed by using RT-qPCR analysis. *U6* was used as an endogenous control to normalize miRNA expression levels. Data are shown as scatter plots and bars overlaid with the mean ± standard error of the mean (SEM). Dot on the plot represents the value of each mouse. Control and sepsis indicate the IECs isolated from sham and sepsis cohorts, respectively. *$P < 0.05$; **$P < 0.01$; and ns, not significant.

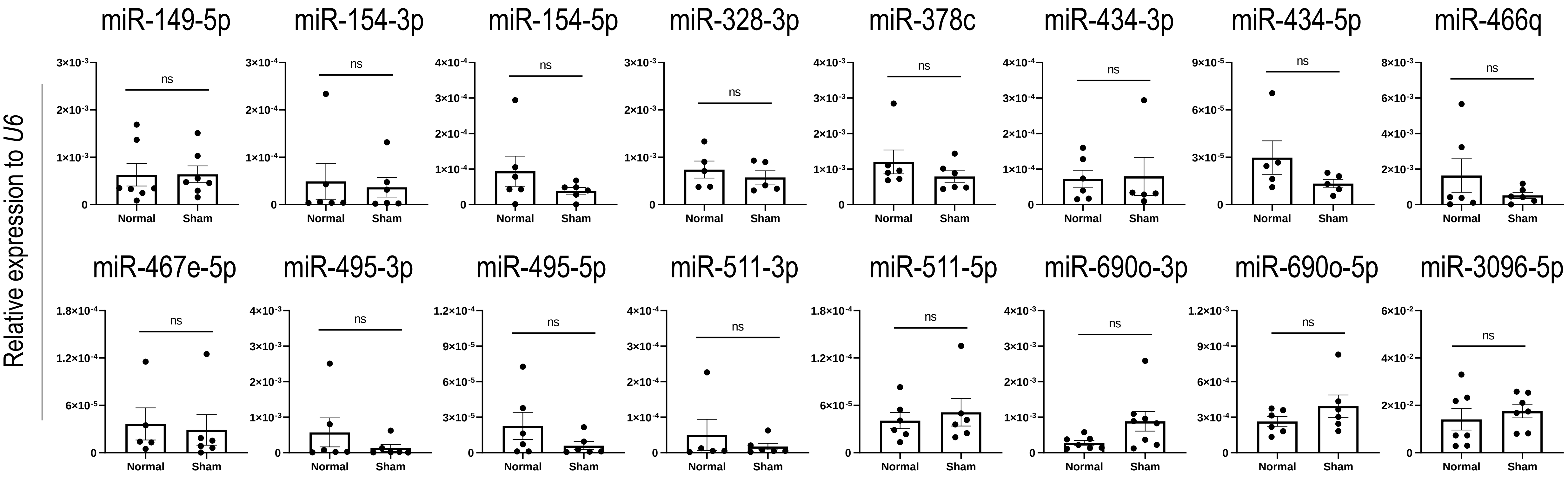

Figure S5. Comparable expression of sepsis-increased miRNAs in IECs of normal and antibiotics-treated sham mice. The miRNAs upregulated in the IECs of sepsis mice were tested for their expressions in the IECs of normal and sham mice by using RT-qPCR analysis. *U6* was used as an endogenous control to normalize miRNA expression levels. Data are shown as scatter plots and bars overlaid with the mean ± standard error of the mean (SEM). Dot on the plot represents the value of each mouse. Control and sepsis indicate the IECs isolated from sham and sepsis cohorts, respectively. ns, not significant.

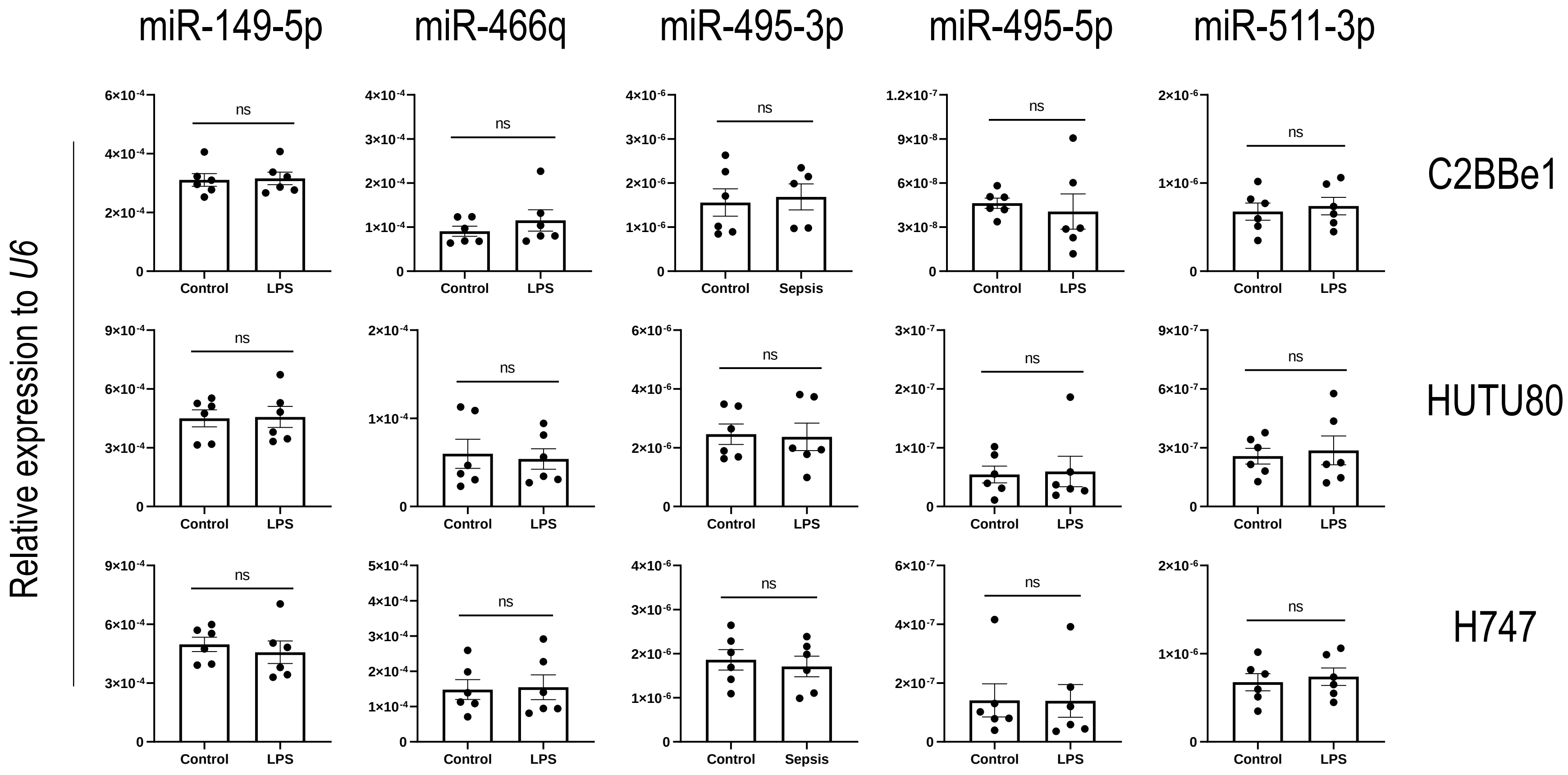

Figure S6. Analysis of expression of the miRNAs upregulated in IECs of sepsis mice in three human IEC lines untreated and treated with LPS. The miRNAs upregulated in the IECs of sepsis mice were tested for their expressions in three human IEC lines (C2BBe1, HUTU80, and H747) between control and LPS treatment by using RT-qPCR analysis. *U6* was used as an endogenous control to normalize miRNA expression levels. Data are shown as scatter plots and bars overlaid with the mean ± standard error of the mean (SEM). Dot on the plot represents the value of each treatment to cells cultured on a well of 6-well plates. Control and LPS indicate the cells untreated and treated with LPS, respectively. ns, not significant.

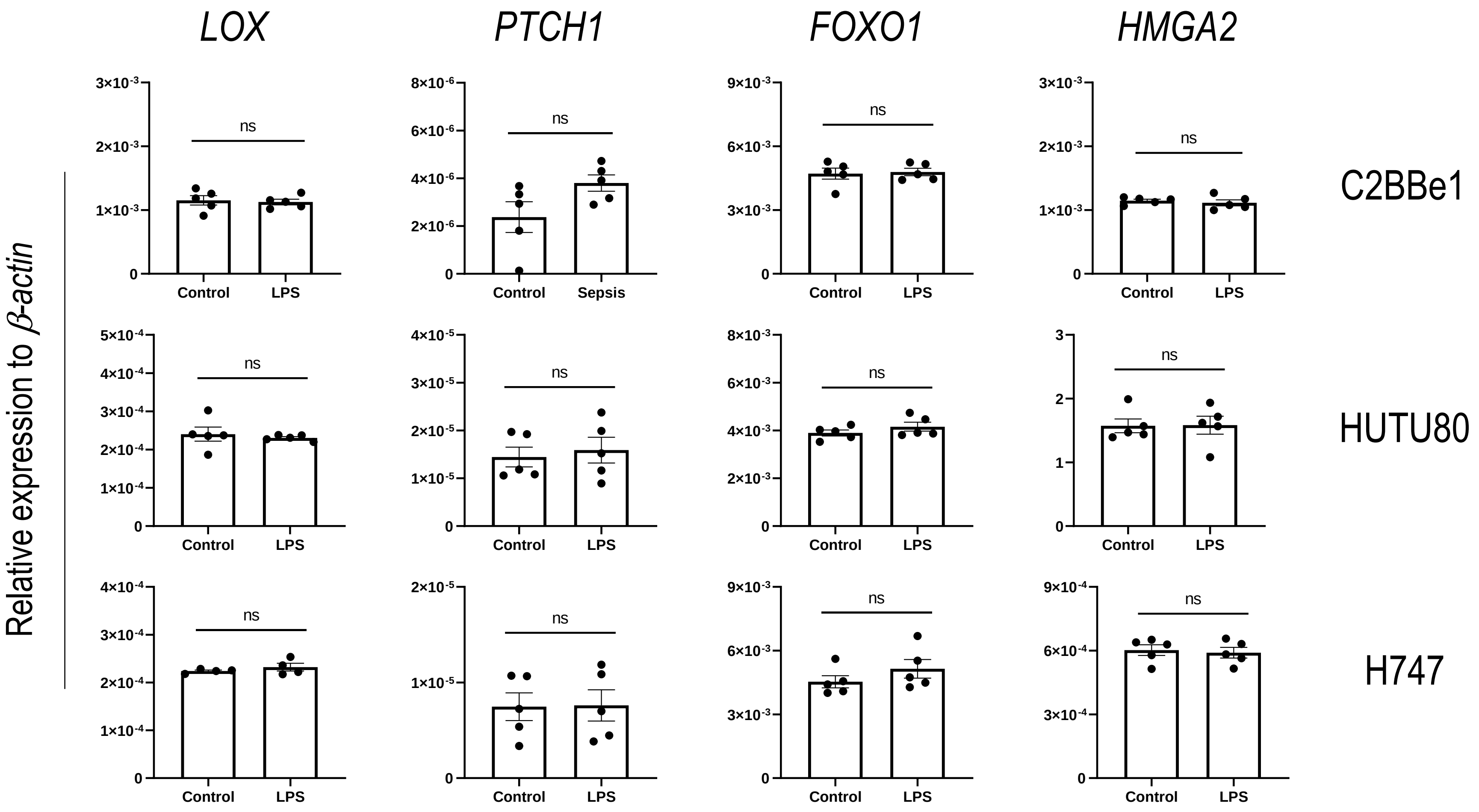

Figure S7. Analysis of expression of four target mRNAs in three human IEC lines untreated and treated with LPS. The expression of the mRNAs of putative targets (LOX, PTCH1, COL22A1, FOXO1, and HMGA2) was analyzed by using RT-qPCR analysis with RNA extracted from the IECs of sham and sepsis mice. *β-actin* was used as an endogenous control to normalize mRNA expression levels. Data are shown as scatter plots and bars overlaid with the mean ± standard error of the mean (SEM). Dot on the plot represents the value of each treatment to cells cultured on a well of 6-well plates. Control and LPS indicate the cells treated with mock (PBS) and LPS, respectively. ns, not significant.

Table S1. List, ENSEMBL No. and RPKM values of all 239 miRNAs detected in IECs

miRNAs	ENSEMBL No.	Sham-IEC	Sepsis1-IEC	Sepsis2-IEC	Sepsis3-IEC
Mir100	ENSMUSG00000093011.1	10.608	12.863	18.25	10.569
Mir101b	ENSMUSG00000065556.1	1778.923	1491.096	1797.01	1215.885
Mir103-1	ENSMUSG00000065553.1	8.486	11.873	12.384	13.652
Mir103-2	ENSMUST00000083629.1	51.978	65.303	61.269	49.322
Mir106a	ENSMUSG00000065456.1	8.486	8.905	13.688	7.046
Mir106b	ENSMUSG00000065514.1	7786.105	7362.472	7592.808	5742.095
Mir107	ENSMUSG00000065594.1	308.686	266.161	365.659	321.476
Mir10a	ENSMUSG00000065519.1	1457.508	2135.226	1480.236	1112.836
Mir10b	ENSMUST00000083566.2	221.702	331.465	245.076	178.794
Mir1198	ENSMUST00000116995.1	11.669	5.937	1.955	1.762
Mir122	ENSMUST00000083468.1	4.243	19.789	8.473	6.606
Mir1224	ENSMUSG00000080669.1	2.122	1.979	1.304	5.725
Mir124-2hg	ENSMUST00000188983.2	4.243	1.979	0.652	0.881
Mir1247	ENSMUSG00000080356.1	1.061	0.989	0.652	0.44
Mir1258	ENSMUSG00000099196.1	19.094	3.958	4.563	3.523
Mir127	ENSMUSG00000070076.1	4.243	30.673	11.732	9.248
Mir128-1	ENSMUST00000083586.1	28.641	21.768	42.367	19.817
Mir130a	ENSMUST00000083550.1	111.382	235.488	174.682	173.069
Mir130b	ENSMUSG00000065572.1	183.514	186.016	192.933	118.021
Mir132	ENSMUST00000083603.1	8.486	13.852	18.25	15.413
Mir135b	ENSMUST00000083501.1	1.061	3.958	2.607	0.881
Mir136	ENSMUSG00000070129.1	10.608	39.578	34.545	17.175
Mir138-1	ENSMUST00000083480.1	4.243	7.916	7.17	6.165
Mir138-2	ENSMUSG00000065512.2	2.122	3.958	0.652	3.523
Mir139	ENSMUST00000083512.1	150.63	162.269	241.166	145.325
Mir140	ENSMUSG00000065439.1	549.483	437.335	634.852	431.571
Mir141	ENSMUST00000083540.1	281.106	278.034	286.14	204.776
Mir143	ENSMUST00000083511.2	2402.66	5340.043	3813.677	3912.763
Mir144	ENSMUSG00000065401.1	5.304	0.989	9.125	1.762
Mir145a	ENSMUST00000083658.1	3303.26	6373.025	5908.559	6315.468
Mir146	ENSMUSG00000070127.2	771.185	1529.684	1192.792	939.768
Mir148a	ENSMUSG00000065505.1	1258.082	1401.056	1499.79	1204.876
Mir148b	ENSMUSG00000065560.1	309.747	402.705	353.275	240.006
Mir149	ENSMUSG00000065470.1	1.061	3.958	2.607	3.523
Mir150	ENSMUSG00000065495.1	110.321	52.441	147.958	120.664
Mir151	ENSMUSG00000065612.1	1142.457	1129.948	1370.734	1009.347
Mir152	ENSMUST00000083581.1	83.801	159.301	163.602	112.297
Mir153	ENSMUSG00000065538.1	19.094	34.631	29.331	16.294
Mir154	ENSMUST00000083514.1	2.122	10.884	14.34	4.844
Mir15a	ENSMUST00000175266.1	4304.634	3287.931	4355.322	2854.093

Mir15b	ENSMUSG00000065580.1	1556.16	400.726	608.129	1047.66
Mir16-1	ENSMUST00000175254.1	118.807	116.755	173.379	83.672
Mir16-2	ENSMUSG00000065606.1	162.299	154.354	184.459	112.737
Mir17hg	ENSMUSG00000089726.2	4.243	1.979	0.652	1.762
Mir181a-1	ENSMUST00000083631.1	7.425	5.937	11.732	3.083
Mir181a-2	ENSMUST00000083489.1	3.182	4.947	1.955	1.321
Mir181b-2	ENSMUST00000083644.1	16.972	25.726	19.554	17.175
Mir181c	ENSMUSG00000065483.1	305.504	423.483	389.776	370.799
Mir181d	ENSMUST00000102383.1	21.216	43.536	34.545	36.992
Mir182	ENSMUSG00000076361.1	557.969	645.119	664.183	428.929
Mir183	ENSMUSG00000065619.2	782.854	683.708	795.847	510.399
Mir185	ENSMUSG00000065464.1	589.792	501.649	634.852	488.38
Mir186	ENSMUST00000083497.1	151.691	238.457	144.699	95.122
Mir187	ENSMUSG00000065532.1	31.823	37.599	44.974	28.625
Mir188	ENSMUSG00000065398.1	24.398	30.673	35.849	24.661
Mir18b	ENSMUST00000103923.2	42.431	53.43	42.367	43.597
Mir190a	ENSMUST00000102424.1	212.155	206.794	211.835	83.232
Mir190b	ENSMUSG00000077996.1	1.061	2.968	2.607	0.881
Mir192	ENSMUSG00000065523.1	22629.561	17348.956	20154.281	13372.533
Mir1927	ENSMUSG00000089204.1	3.182	2.968	3.259	2.642
Mir1934	ENSMUST00000158127.1	1.061	3.958	1.955	2.642
Mir1938	ENSMUSG00000089371.1	2.122	0.989	0.652	1.321
Mir193a	ENSMUST00000083461.1	49.857	105.871	56.707	60.332
Mir193b	ENSMUST00000103784.2	3.182	6.926	4.563	9.248
Mir194-1	ENSMUST00000083647.1	5424.815	2457.785	4372.269	3444.2
Mir194-2	ENSMUSG00000065582.1	6173.723	4725.597	5503.14	4886.88
Mir195a	ENSMUSG00000065411.2	51.978	85.092	74.305	73.984
Mir1964	ENSMUST00000158514.1	3.182	5.937	4.563	4.404
Mir1968	ENSMUST00000157429.1	8.486	9.894	11.732	11.89
Mir196a-1	ENSMUSG00000065546.1	2.122	1.979	3.911	2.642
Mir196a-2	ENSMUSG00000065488.1	21.216	20.778	9.777	9.688
Mir1981	ENSMUSG00000088559.1	31.823	17.81	30.635	36.551
Mir1983	ENSMUST00000157523.1	319.294	150.396	153.173	121.104
Mir199b	ENSMUSG00000092807.1	8.486	26.715	18.25	9.688
Mir19b-2	ENSMUST00000083539.1	117.746	140.501	166.209	110.095
Mir200a	ENSMUST00000083466.1	44587.649	45418.566	47080.103	32250.678
Mir200b	ENSMUST00000083615.2	23220.414	21002.982	21854.825	14951.73
Mir200c	ENSMUSG00000065462.2	11919.954	15122.701	12494.337	9800.183
Mir203	ENSMUSG00000065574.1	4804.26	4863.13	5416.451	3354.363
Mir20b	ENSMUST00000102087.1	141.083	143.47	118.627	133.435
Mir210	ENSMUSG00000065551.1	55.16	79.156	51.492	40.515
Mir212	ENSMUST00000083656.1	2.122	7.916	10.429	2.642
Mir2137	ENSMUSG00000089357.1	1.061	0.989	0.652	1.321

Mir21c	ENSMUSG00000099326.1	25.459	26.715	29.331	10.569
Mir221	ENSMUSG00000065422.1	212.155	278.034	321.337	206.538
Mir222	ENSMUST00000083537.1	155.934	161.28	194.236	132.554
Mir224	ENSMUSG00000065542.1	113.503	93.997	114.717	59.011
Mir22hg	ENSMUSG00000085148.1	8.486	4.947	9.777	7.046
Mir23a	ENSMUSG00000065611.1	729.815	763.853	1173.89	610.365
Mir23b	ENSMUSG00000065599.1	3581.184	3099.936	4194.979	2365.713
Mir24-2	ENSMUST00000083607.1	21.216	22.757	24.117	16.734
Mir26a-1	ENSMUST00000083579.1	31.823	24.736	35.849	14.973
Mir26a-2	ENSMUSG00000065430.1	9.547	9.894	20.858	9.688
Mir26b	ENSMUST00000083534.1	2175.654	1449.539	1903.905	1096.102
Mir27a	ENSMUST00000083510.1	447.648	620.383	731.97	364.193
Mir27b	ENSMUST00000083541.1	3091.105	3446.242	3619.441	1925.335
Mir28a	ENSMUSG00000065494.1	393.548	323.549	348.713	305.182
Mir296	ENSMUST00000083587.1	21.216	40.567	10.429	35.23
Mir298	ENSMUSG00000065410.1	28.641	43.536	11.732	33.469
Mir29a	ENSMUST00000083676.1	9611.702	11299.48	11057.12	7502.728
Mir29b-1	ENSMUST00000083670.1	18.033	16.821	27.376	17.175
Mir300	ENSMUST00000083485.2	4.243	12.863	10.429	5.285
Mir301	ENSMUSG00000065589.1	434.919	276.056	331.114	254.539
Mir301b	ENSMUSG00000076288.1	39.249	52.441	46.278	25.542
Mir3057	ENSMUSG00000092781.1	1.061	3.958	0.652	0.44
Mir3060	ENSMUSG00000093080.1	1.061	0.989	1.304	3.083
Mir3074-1	ENSMUSG00000092741.1	1.061	0.989	0.652	1.762
Mir3076	ENSMUSG00000093156.1	1.061	0.989	0.652	0.881
Mir3091	ENSMUST00000175086.1	2.122	1.979	7.17	2.642
Mir3095	ENSMUSG00000093293.1	1.061	1.979	1.955	3.963
Mir3096	ENSMUST00000116685.1	4.243	34.631	12.384	27.303
Mir30a	ENSMUSG00000065405.2	2055.786	2307.389	2762.977	1835.938
Mir30b	ENSMUST00000083542.2	7201.617	7394.134	8320.868	5099.142
Mir30c-1	ENSMUSG00000065490.1	40.31	20.778	24.117	19.377
Mir30e	ENSMUSG00000065409.1	3232.188	3093.01	3588.154	2350.74
Mir31	ENSMUSG00000065408.1	6531.205	5004.621	6892.124	4821.264
Mir3105	ENSMUSG00000093221.1	56.221	48.483	66.484	60.332
Mir3109	ENSMUSG00000093042.1	1.061	1.979	0.652	0.881
Mir3110	ENSMUST00000175053.1	2.122	0.989	1.304	0.44
Mir32	ENSMUSG00000065544.1	849.683	563.985	545.556	339.532
Mir320	ENSMUSG00000065528.1	295.957	446.24	394.338	359.789
Mir322	ENSMUSG00000065418.1	16.972	13.852	27.376	19.817
Mir326	ENSMUST00000083637.1	61.525	93.008	66.484	48.442
Mir328	ENSMUST00000093622.1	3.182	17.81	11.081	8.367
Mir329	ENSMUST00000083643.1	3.182	6.926	14.991	2.642
Mir33	ENSMUST00000083531.1	877.263	1077.507	795.195	550.913

Mir337	ENSMUSG00000065526.3	5.304	8.905	9.777	5.285
Mir338	ENSMUSG00000065600.1	5.304	5.937	9.777	2.642
Mir339	ENSMUST00000083659.1	152.752	159.301	204.013	127.269
Mir340	ENSMUSG00000065417.1	212.155	283.971	314.167	151.05
Mir342	ENSMUSG00000065436.1	152.752	180.079	193.584	141.802
Mir345	ENSMUST00000083495.1	808.312	794.526	860.375	571.171
Mir3473d	ENSMUST00000175346.1	7.425	1.979	6.518	3.523
Mir34a	ENSMUST00000083559.1	526.146	669.855	782.811	754.368
Mir34b	ENSMUST00000083558.1	7.425	8.905	11.732	3.523
Mir34c	ENSMUSG00000065587.1	1.061	6.926	8.473	1.762
Mir350	ENSMUSG00000065573.1	32.884	25.726	54.099	16.734
Mir351	ENSMUSG00000065503.1	2.122	0.989	0.652	0.881
Mir3547	ENSMUSG00000093202.2	3.182	1.979	1.304	2.202
Mir361	ENSMUSG00000065510.1	275.802	192.942	266.586	215.345
Mir362	ENSMUSG00000099172.1	219.581	248.351	244.425	144.004
Mir363	ENSMUSG00000070106.1	81.68	68.272	70.394	74.424
Mir369	ENSMUST00000083627.2	6.365	7.916	3.259	3.083
Mir374b	ENSMUST00000102314.1	844.379	738.127	840.821	601.117
Mir375	ENSMUST00000083682.2	2577.689	3507.588	3760.229	2300.978
Mir376b	ENSMUSG00000076006.1	4.243	10.884	6.518	6.165
Mir378c	ENSMUST00000184478.1	1.061	3.958	3.259	3.523
Mir379	ENSMUSG00000065498.3	8.486	25.726	24.117	14.973
Mir382	ENSMUSG00000065428.3	2.122	7.916	3.259	3.963
Mir411	ENSMUSG00000065477.2	6.365	18.799	22.813	4.404
Mir421	ENSMUST00000083575.2	31.823	24.736	29.331	21.579
Mir423	ENSMUSG00000065518.2	553.726	812.336	804.972	659.687
Mir431	ENSMUSG00000070080.1	1.061	2.968	1.955	2.202
Mir434	ENSMUSG00000070133.1	5.304	32.652	28.679	16.734
Mir449a	ENSMUST00000083641.1	5.304	11.873	13.036	7.927
Mir449c	ENSMUSG00000076146.1	3.182	2.968	1.304	1.762
Mir451a	ENSMUSG00000070065.1	13.79	45.515	48.233	21.579
Mir452	ENSMUSG00000070138.1	2.122	1.979	2.607	1.321
Mir455	ENSMUST00000093594.1	13.79	19.789	7.17	14.532
Mir466b-2	ENSMUSG00000076966.2	2.122	0.989	1.304	1.762
Mir466q	ENSMUST00000175249.1	1.061	5.937	7.17	4.844
Mir467a-1	ENSMUSG00000096624.1	2.122	2.968	3.259	3.083
Mir467a-10	ENSMUST00000179320.1	3.182	8.905	1.955	2.642
Mir467a-2	ENSMUST00000179447.1	1.061	0.989	1.955	2.202
Mir467a-3	ENSMUST00000178082.1	1.061	2.968	1.955	0.44
Mir467a-7	ENSMUSG00000094688.1	2.122	0.989	1.304	0.881
Mir467c	ENSMUSG00000077049.2	3.182	1.979	8.473	3.523
Mir467d	ENSMUST00000103833.2	1.061	3.958	1.955	0.881
Mir467e	ENSMUST00000103760.2	2.122	15.831	11.081	5.285

Mir484	ENSMUST00000093566.1	819.981	1078.497	978.35	845.086
Mir490	ENSMUSG00000070075.1	2.122	0.989	2.607	2.642
Mir491	ENSMUST00000093569.2	2.122	3.958	1.955	0.44
Mir494	ENSMUSG00000070141.2	5.304	3.958	3.259	0.881
Mir495	ENSMUSG00000070105.2	1.061	9.894	3.911	3.083
Mir500	ENSMUSG00000070108.2	85.923	83.114	99.073	60.332
Mir501	ENSMUST00000102296.1	19.094	34.631	20.206	14.973
Mir503	ENSMUST00000102168.1	21.216	12.863	20.858	19.377
Mir505	ENSMUST00000093573.2	23.337	20.778	28.027	13.652
Mir5099	ENSMUSG00000092998.1	82.741	82.124	90.6	59.891
Mir5100	ENSMUSG00000092734.1	10.608	11.873	26.724	7.927
Mir511	ENSMUST00000104704.1	1.061	11.873	2.607	2.642
Mir5125	ENSMUSG00000092981.1	4.243	1.979	1.304	1.321
Mir5126	ENSMUST00000175513.1	1.061	0.989	4.563	0.44
Mir5136	ENSMUST00000175573.1	1.061	2.968	1.304	0.881
Mir532	ENSMUSG00000070139.1	15.912	19.789	22.813	12.331
Mir541	ENSMUST00000102097.1	3.182	4.947	8.473	3.083
Mir542	ENSMUST00000102186.1	1.061	0.989	2.607	3.523
Mir574	ENSMUSG00000077042.2	32.884	42.546	60.617	28.625
Mir582	ENSMUST00000103758.2	329.902	280.013	329.159	173.509
Mir598	ENSMUST00000102094.3	11.669	4.947	1.955	6.606
Mir615	ENSMUST00000102056.1	1.061	1.979	1.304	0.881
Mir6236	ENSMUSG00000098973.1	616.312	798.483	374.784	917.308
Mir6238	ENSMUSG00000099176.1	173.967	29.683	37.153	40.515
Mir6239	ENSMUSG00000098648.1	377.637	315.633	104.94	274.796
Mir6240	ENSMUSG00000098343.1	1080.932	314.644	481.68	446.984
Mir6406	ENSMUSG00000099174.1	2.122	0.989	4.563	2.642
Mir652	ENSMUSG00000076011.1	456.134	399.736	437.357	395.46
Mir6538	ENSMUST00000183713.1	93.348	56.398	54.099	46.24
Mir6539	ENSMUST00000183732.1	3.182	1.979	7.822	5.285
Mir669a-2	ENSMUST00000102060.1	5.304	5.937	5.866	0.881
Mir669a-4	ENSMUST00000178277.1	2.122	11.873	11.732	4.404
Mir669a-5	ENSMUSG00000094547.1	1.061	3.958	3.911	3.083
Mir669a-8	ENSMUSG00000096602.1	2.122	3.958	4.563	3.083
Mir669a-9	ENSMUSG00000095225.1	2.122	1.979	0.652	1.762
Mir669c	ENSMUSG00000076118.1	1.061	6.926	3.911	1.762
Mir669o	ENSMUSG00000077086.1	1.061	8.905	5.866	4.844
Mir672	ENSMUST00000102331.1	5.304	14.842	1.304	4.404
Mir674	ENSMUST00000102421.1	160.177	158.311	185.111	121.985
Mir677	ENSMUSG00000093245.1	162.299	155.343	76.912	167.784
Mir6946	ENSMUST00000183515.1	1.061	1.979	1.304	1.321
Mir6969	ENSMUST00000183777.1	3.182	4.947	6.518	3.523
Mir7-1	ENSMUSG00000065434.1	242.918	273.087	293.31	176.592

Mir7-2	ENSMUSG00000065609.1	27.58	28.694	38.456	19.817
Mir700	ENSMUST00000102169.1	29.702	39.578	24.117	19.377
Mir7003	ENSMUSG00000099091.1	7.425	4.947	5.214	7.046
Mir7043	ENSMUST00000184219.1	3.182	6.926	4.563	3.523
Mir708	ENSMUST00000102189.1	2.122	4.947	2.607	2.202
Mir717	ENSMUSG00000076214.1	5.304	1.979	2.607	2.202
Mir744	ENSMUST00000103261.1	230.189	290.897	232.04	154.573
Mir7662	ENSMUST00000184761.1	2.122	3.958	3.259	1.762
Mir7669	ENSMUST00000183442.1	5.304	4.947	1.955	1.321
Mir7b	ENSMUST00000083557.1	89.105	81.135	102.332	73.103
Mir802	ENSMUSG00000076457.1	3731.814	2267.812	3191.209	1680.044
Mir8103	ENSMUST00000183945.1	3.182	6.926	3.911	1.321
Mir872	ENSMUST00000104728.1	79.558	98.945	88.645	79.709
Mir877	ENSMUST00000104738.1	46.674	61.346	65.18	35.23
Mir92-2	ENSMUST00000083679.1	42.431	77.177	48.233	36.111
Mir96	ENSMUSG00000065586.1	218.52	225.594	224.871	169.986
Mir98	ENSMUST00000083602.2	832.71	739.117	829.74	520.527
Mir99a	ENSMUSG00000065530.1	41.37	81.135	52.144	55.488
Mir99b	ENSMUST00000083462.1	9.547	22.757	13.688	11.009
Mirlet7a-1	ENSMUSG00000065421.2	23.337	27.705	35.197	22.459
Mirlet7a-2	ENSMUSG00000092770.1	21.216	8.905	19.554	20.257
Mirlet7b	ENSMUST00000083630.1	3258.708	4874.014	5181.803	3682.004
Mirlet7c-1	ENSMUSG00000065557.2	29.702	31.662	31.286	30.826
Mirlet7c-2	ENSMUST00000083674.1	39.249	55.409	59.314	52.405
Mirlet7f-2	ENSMUST00000083668.1	301.261	298.813	427.58	261.585
Mirlet7g	ENSMUSG00000065440.1	9988.278	9117.75	11845.146	7639.686
Mirlet7i	ENSMUST00000083472.2	3361.603	3387.865	3816.284	2434.412

Table S2. Primer sequences for RT-qPCR used in this study

	Forward (5' → 3')	Reverse (5' → 3')
mmu-miR-127-3p	TCGGATCCGTCTGAGCTTGGCT	Universal primer
mmu-miR-149-5p	TCTGGCTCCGTGTCTTCACTCCC	Universal primer
mmu-miR-154-3p	AATCATACACGGTTGACCTATT	Universal primer
mmu-miR-154-5p	TAGGTTATCCGTGTTGCCTTCG	Universal primer
mmu-miR-328-3p	CTGGCCCTCTCTGCCCTTCCGT	Universal primer
mmu-miR-351-5p	TCCCTGAGGAGCCCTTTGAGCCTG	Universal primer
mmu-miR-378c	ACTGGACTTGGAGTCAGAAGC	Universal primer
mmu-miR-434-3p	TTTGAACCATCACTCGACTCCT	Universal primer
mmu-miR-434-5p	GCTCGACTCATGGTTTGAACCA	Universal primer
mmu-miR-466q	GTGCACACACACACATACGT	Universal primer
mmu-miR-467e-5p	ATAAGTGTGAGCATGTATATGT	Universal primer
mmu-miR-495-3p	GAAGTTGCCCATGTTATTTTTC	Universal primer
mmu-miR-495-5p	GAAGTTGCCCATGTTATTTTTCG	Universal primer
mmu-miR-511-3p	AATGTGTAGCAAAAGACAGGAT	Universal primer
mmu-miR-511-5p	ATGCCTTTTGCTCTGCACTCA	Universal primer
mmu-miR-669o-3p	ACATAACATACACACACACGTAT	Universal primer
mmu-miR-669o-5p	TAGTTGTGTGTGCATGTTTATGT	Universal primer
mmu-miR-717	TAGTTGTGTGTGCATGTTTATGT	Universal primer
mmu-miR-1258-3p	TTAGGGAATTAGCTCAGCAGTA	Universal primer
mmu-miR-1983	CTCACCTGGAGCATGTTTTCT	Universal primer
mmu-miR-3096-5p	TTGGCCAAGGATGAGAAC	Universal primer
mmu-miR-5125	TCTGCCTGGGATTTCCTTGT	Universal primer
hsa-miR-149-5p	TCTGGCTCCGTGTCTTCACTCCC	Universal primer
hsa-miR-466q	GTGCACACACACACATACGT	Universal primer
has-miR-495-3p	GAAGTTGCCCATGTTATTTTTC	Universal primer
has-miR-495-5p	GAAGTTGCCCATGTTATTTTTCG	Universal primer
hsa-miR-511-3p	AATGTGTAGCAAAAGACAGA	Universal primer
U6	GCGCGTCGTGAAGCGTTC	GTGCAGGGTCCGAGGT
mouse DNMT1	CCTAGTTCCGTGGCTACGAGGAGAA	TCTCTCTCCTCTGCAGCCGACTCA
mouse DNMT3A	GCCGAATTGTGTCTTGGTGGATGACA	CCTGGTGGAATGCACTGCAGAAGGA
mouse LOX	AAGCAGAGCCTTCCTGCAAA	GGTCACAGCGGTCTCGTTGT
mouse PTCH1	CGGACCGGGACTATCTGCAC	CCTTCCCCTTGGAAATCTGCT
mouse COL22A1	GGGGAACCTGGATACGCTAAA	CAAAGTACGCACACTGGGAG

mouse FOXO1	ACATTTCGTCCTCGAACCAGCTCA	ATTTCAGACAGACTGGGCAGCGTA
mouse HMGA2	TGAGTTTGGAGAACGCACCA	TGAGAGTGGAAGCGATGAGC
mouse β-actin	CATCGTACTCCTGCTTGCTG	AGCGCAAGTACTCTGTGTGG
human LOX	GCAGATGTCAGAGATTATGATCA	ATCGCCTGTGGTAGCCATAGT
human PTCH1	GTTGCAGCGTTAAAGGAA	CCAGCGGCTACTTACTCA
human FOXO1	AGACAACGACACATAGCTGG	AGGGAGTTGGTGAAAGACATC
human HMGA2	GGCTCAGATTCAGGAACAGC	GCTTCAACGGCAAAGTTCTC
human β-actin	GGATGCAGAAGGAGATCACTG	CGATCCACACGGAGTACTTG

Table S3. Reactome pathways detected in IECs with sepsis, hit numbers, and *P* values

Name	Hits	P value	Adjusted P value
Innate immune system	42	0.00408	0.0075556
Developmental biology	37	0.0000377	0.0009529
Signalling by NGF	28	0.0000982	0.0009529
Axon guidance	28	0.000162	0.0009529
Cytokine signaling in immune system	23	0.0159	0.0172826
Fc epsilon receptor (FCERI) signaling	20	0.0000789	0.0009529
NGF signalling via TRKA from the plasma membrane	20	0.000365	0.001587
Signaling by PDGF	19	0.000407	0.0016958
Downstream signal transduction	18	0.000138	0.0009529
Signaling by FGFR1	18	0.000138	0.0009529
Signaling by FGFR	18	0.000138	0.0009529
Signaling by FGFR2	18	0.000138	0.0009529
Signaling by FGFR3	18	0.000138	0.0009529
Signaling by FGFR4	18	0.000138	0.0009529
Signaling by ERBB2	18	0.000151	0.0009529
DAP12 signaling	18	0.000235	0.0011381
Signaling by EGFR	18	0.000569	0.0020944
DAP12 interactions	18	0.000613	0.0020944
Signaling by ERBB4	18	0.000712	0.0020944
Downstream signaling of activated FGFR1	17	0.000144	0.0009529
Downstream signaling of activated FGFR2	17	0.000144	0.0009529
Downstream signaling of activated FGFR3	17	0.000144	0.0009529
Downstream signaling of activated FGFR4	17	0.000144	0.0009529
Downstream signaling events of B cell receptor (BCR)	17	0.00124	0.003
Signaling by the B cell receptor (BCR)	17	0.0066	0.0101539
Generic transcription pathway	17	0.00774	0.01075
Gastrin-CREB signalling pathway via PKC and MAPK	17	0.00815	0.0110135
Transmission across chemical synapses	17	0.0095	0.0123205
Signaling by SCF-KIT	15	0.000962	0.0025316
Apoptosis	15	0.00812	0.0110135
Programmed cell death	15	0.00961	0.0123205
Signaling by interleukins	14	0.000602	0.0020944
Neurotransmitter receptor binding and downstream transmission in the postsynaptic cell	14	0.00418	0.0076
Membrane trafficking	14	0.0113	0.0134524
IRS-related events triggered by IGF1R	13	0.00016	0.0009529
IGF1R signaling cascade	13	0.00023	0.0011381
Signaling by type 1 insulin-like growth factor 1 receptor (IGF1R)	13	0.00023	0.0011381
PI-3K cascade:FGFR1	12	0.000754	0.0020944
PI-3K cascade:FGFR2	12	0.000754	0.0020944
PI-3K cascade:FGFR3	12	0.000754	0.0020944
PI-3K cascade:FGFR4	12	0.000754	0.0020944
PIP3 activates AKT signaling	12	0.000754	0.0020944

PI3K events in ERBB4 signaling	12	0.000754	0.0020944
PI3K events in ERBB2 signaling	12	0.000754	0.0020944
GAB1 signalosome	12	0.00114	0.00285
PI3K/AKT activation	12	0.00114	0.00285
Role of LAT2/NTAL/LAB on calcium mobilization	12	0.00201	0.0043696
Signaling by VEGF	12	0.00284	0.0059167
IRS-mediated signalling	11	0.00141	0.0032791
IRS-related events	11	0.0019	0.0043182
Insulin receptor signalling cascade	11	0.00305	0.0062245
VEGFA-VEGFR2 pathway	11	0.00432	0.0077143
Signaling by insulin receptor	11	0.0181	0.0182828
Translocation of Glut4 to the plasma membrane	10	0.000239	0.0011381
Translocation of GLUT4 to the plasma membrane	10	0.000279	0.0012682
Transport of inorganic cations/anions and amino acids/oligopeptides	10	0.0174	0.0179592
TP53 regulates metabolic genes	8	0.00605	0.0094531
Transcriptional regulation by TP53	8	0.00605	0.0094531
GABA receptor activation	8	0.00744	0.0104789
PI3K cascade	8	0.0168	0.0178723
Caspase activation via extrinsic apoptotic signalig pathway	7	0.0000935	0.0009529
Nucleotide-binding domain, leucine rich repeat containing receptor (NLR) signaling pathways	7	0.00397	0.0074906
Interleukin-1 signaling	7	0.00515	0.0085833
Amino acid and oligopeptide SLC transporters	7	0.0103	0.0124096
G alpha (12/13) signalling events	7	0.0184	0.0184
Activation of GABAB receptors	6	0.0176	0.0179592
GABA B receptor activation	6	0.0176	0.0179592
CD28 dependent PI3K/Akt signaling	5	0.000496	0.001984
Frs2-mediated activation	5	0.00126	0.003
Prolonged ERK activation events	5	0.0021	0.0044681
NOD1/2 signaling pathway	5	0.00704	0.0104789
CD28 co-stimulation	5	0.00704	0.0104789
Synthesis of IP3 and IP4 in the cytosol	5	0.00831	0.01108
FRS-mediated FGFR1 signaling	5	0.013	0.0152941
Formation of transcription-coupled NER (TC-NER) repair complex	5	0.015	0.0164835
Dual incision reaction in TC-NER	5	0.015	0.0164835
Ca2+ pathway	5	0.017	0.0178947
Pre-NOTCH expression and processing	4	0.000867	0.0023432
Signalling to p38 via RIT and RIN	4	0.00465	0.0081579
Ligand-dependent caspase activation	4	0.00597	0.0094531
ARMS-mediated activation	4	0.00597	0.0094531
SHC-related events triggered by IGF1R	4	0.00931	0.01225
JNK (c-Jun kinases) phosphorylation and activation mediated by activated human TAK1	4	0.0137	0.0153933
Signaling by leptin	4	0.0163	0.0175269
Beta-catenin phosphorylation cascade	3	0.00196	0.0043556
MEK activation	3	0.00331	0.0063654

RAF phosphorylates MEK	3	0.00331	0.0063654
Post-transcriptional silencing by small RNAs	3	0.00331	0.0063654
Ligand-independent caspase activation via DCC	3	0.00511	0.0085833
Netrin mediated repulsion signals	3	0.00511	0.0085833
Adrenoceptors	3	0.0074	0.0104789
Sodium/calcium exchangers	3	0.0074	0.0104789
HuR stabilizes mRNA	3	0.0074	0.0104789
RAF/MAP kinase cascade	3	0.0102	0.0124096
Processing and activation of SUMO	3	0.0102	0.0124096
Regulation by c-FLIP	3	0.0102	0.0124096
CASP8 activity is inhibited	3	0.0102	0.0124096
NF-kB activation through FADD/RIP-1 pathway mediated by caspase-8 and -10	3	0.0135	0.0153409
AKT phosphorylates targets in the cytosol	3	0.0135	0.0153409
Calnexin/calreticulin cycle	3	0.0135	0.0153409

Table S4. GO pathways detected in IECs with sepsis, hit numbers, and *P* values

Name	Hits	P value	Adjusted P value
Regulation of signal transduction	139	0.00104	0.0049524
Intracellular signal transduction	135	0.000617	0.0032474
Nervous system development	133	0.00427	0.0090408
Regulation of molecular function	132	0.0113	0.0137805
Cell development	117	0.00875	0.0123239
Regulation of cellular component organization	108	0.000044	0.0008856
Negative regulation of cellular metabolic process	107	0.00923	0.0126438
Neurogenesis	103	0.0000694	0.0008856
Generation of neurons	97	0.0000887	0.000887
Regulation of cellular protein metabolic process	88	0.00763	0.0118235
Neuron differentiation	81	0.002	0.0068
Intracellular protein kinase cascade	73	0.00221	0.0068
Neuron development	70	0.0000549	0.0008856
Regulation of protein modification process	70	0.0097	0.0129114
Positive regulation of signal transduction	64	0.00764	0.0118235
Cellular nitrogen compound catabolic process	63	0.0167	0.017579
Neuron projection development	60	0.000119	0.0010818
Positive regulation of catalytic activity	60	0.00234	0.0068
Positive regulation of protein metabolic process	60	0.00914	0.0126438
Positive regulation of cellular component organization	58	6.22E-06	0.000622
Transmission of nerve impulse	56	0.000925	0.004625
Cell morphogenesis involved in differentiation	54	0.000393	0.0023118
Central nervous system development	53	0.00245	0.0068056
Positive regulation of hydrolase activity	53	0.0171	0.0178125
Regulation of anatomical structure morphogenesis	52	0.0136	0.0156322
Positive regulation of cellular protein metabolic process	52	0.0156	0.0169565
Regulation of neurogenesis	51	0.000078	0.0008856
Behavior	51	0.00192	0.0068
Negative regulation of transcription from RNA polymerase II promoter	51	0.0102	0.0129114
Negative regulation of programmed cell death	51	0.0142	0.0161364
Regulation of catabolic process	50	0.0000644	0.0008856
Negative regulation of apoptotic process	50	0.0165	0.0175532
Cell-cell signaling	50	0.0165	0.0175532
Positive regulation of phosphate metabolic process	49	0.00599	0.0100333
Positive regulation of protein modification process	48	0.0101	0.0129114
Brain development	47	0.000054	0.0008856
Synaptic transmission	45	0.00359	0.0081591
MAPK cascade	45	0.00736	0.0118235
Positive regulation of phosphorylation	43	0.00661	0.0108361
Regulation of MAPK cascade	42	0.00297	0.0072439
Positive regulation of protein phosphorylation	41	0.0101	0.0129114
Negative regulation of protein metabolic process	40	0.00136	0.0061818

Negative regulation of cellular protein metabolic process	38	0.000215	0.0016538
Axonogenesis	38	0.00026	0.0018571
Small GTPase mediated signal transduction	38	0.00238	0.0068
I-kappaB kinase/NF-kappaB cascade	36	0.0177	0.0179798
Regulation of cell morphogenesis	33	0.000333	0.0020813
Regulation of membrane potential	31	0.00275	0.0070513
Regulation of small GTPase mediated signal transduction	30	0.00333	0.0077442
Positive regulation of MAPK cascade	30	0.00391	0.0086889
Cell-cell adhesion	30	0.00572	0.0100333
Regulation of Ras protein signal transduction	28	0.002	0.0068
Regulation of cell adhesion	27	0.00193	0.0068
Transmembrane receptor protein tyrosine kinase signaling pathway	27	0.0174	0.0179381
Negative regulation of transport	26	0.0135	0.0156322
Learning or memory	25	0.0000131	0.000655
Ras protein signal transduction	25	0.000284	0.0018933
Negative regulation of phosphate metabolic process	25	0.00804	0.0118235
Regulation of neuron apoptotic process	23	0.000206	0.0016538
Regulation of MAP kinase activity	23	0.00234	0.0068
Cellular carbohydrate metabolic process	23	0.00443	0.0090408
Wound healing	23	0.00822	0.011913
Regulation of body fluid levels	23	0.0109	0.0134568
Neuron apoptotic process	22	0.00166	0.0068
Regulation of GTPase activity	22	0.0027	0.0070513
Glycoprotein metabolic process	22	0.00844	0.0120571
Apoptotic signaling pathway	21	0.00789	0.0118235
Glycoprotein biosynthetic process	20	0.00573	0.0100333
Cell-substrate adhesion	20	0.00602	0.0100333
Regulation of Ras GTPase activity	19	0.00196	0.0068
Locomotory behavior	19	0.00235	0.0068
Regulation of action potential	19	0.00333	0.0077442
Axon guidance	19	0.00415	0.0090217
Gliogenesis	19	0.00601	0.0100333
Positive regulation of MAP kinase activity	18	0.00236	0.0068
Response to toxin	18	0.0178	0.0179798
Synapse organization	17	0.00235	0.0068
Amino acid transport	17	0.00551	0.0100333
Regulation of JNK cascade	16	0.00564	0.0100333
Regulation of Rho protein signal transduction	16	0.0109	0.0134568
Blood coagulation	15	0.0102	0.0129114
Coagulation	15	0.0148	0.0162637
Protein polyubiquitination	14	0.00436	0.0090408
Regulation of axonogenesis	14	0.00534	0.0100333
Response to hypoxia	14	0.0148	0.0162637
Synapse assembly	13	0.0000797	0.0008856
Cell-matrix adhesion	13	0.0145	0.0162637
G1/S transition of mitotic cell cycle	12	0.00775	0.0118235

Regulation of synapse structure and activity	11	0.000599	0.0032474
Glucan metabolic process	11	0.0027	0.0070513
Regulation of cell-cell adhesion	11	0.0046	0.0092
Energy reserve metabolic process	11	0.00543	0.0100333
Cellular polysaccharide metabolic process	11	0.00803	0.0118235
Polysaccharide metabolic process	11	0.0132	0.0155294
Peripheral nervous system development	9	0.00293	0.0072439
Positive regulation of JUN kinase activity	8	0.0121	0.0145238
Protein export from nucleus	7	0.00569	0.0100333
Post-golgi vesicle-mediated transport	6	0.0122	0.0145238
Ectoderm development	4	0.00987	0.0129114
Calnexin/calreticulin cycle	3	0.0135	0.0153409

Table S5. KEGG pathways detected in IECs with sepsis, hit numbers, and *P* values

Name	Hits	P value	Adjusted P value
Pathways in cancer	33	0.000478	0.0053111
Proteoglycans in cancer	21	0.000367	0.0046875
MicroRNAs in cancer	21	0.0133	0.0271429
Axon guidance	20	1.15E-06	0.000115
Rap1 signaling pathway	20	0.00159	0.0083684
MAPK signaling pathway	20	0.0105	0.0238636
FoxO signaling pathway	18	0.0000304	0.00152
Chemokine signaling pathway	18	0.00344	0.0132308
Oxytocin signaling pathway	17	0.000756	0.0054071
cGMP-PKG signaling pathway	17	0.00194	0.0092381
Dopaminergic synapse	16	0.000367	0.0046875
Wnt signaling pathway	16	0.000757	0.0054071
Calcium signaling pathway	16	0.00735	0.0204167
Neurotrophin signaling pathway	15	0.000375	0.0046875
Signaling pathways regulating pluripotency of stem cells	15	0.00159	0.0083684
Hippo signaling pathway	15	0.00403	0.0143929
Melanogenesis	14	0.000133	0.0044333
Chagas disease (American trypanosomiasis)	14	0.000204	0.0046875
Insulin signaling pathway	14	0.00454	0.0151667
Phospholipase D signaling pathway	14	0.00547	0.0165588
Hepatitis B	14	0.00618	0.0176571
Adrenergic signaling in cardiomyocytes	14	0.00874	0.0217317
Measles	13	0.00891	0.0217317
Prostate cancer	12	0.00062	0.0054071
Retrograde endocannabinoid signaling	12	0.00226	0.0098261
Glutamatergic synapse	12	0.00563	0.0165588
AMPK signaling pathway	12	0.0136	0.0272
Serotonergic synapse	12	0.017	0.0320755
ErbB signaling pathway	11	0.00177	0.00885
Circadian entrainment	11	0.00455	0.0151667
T cell receptor signaling pathway	11	0.00765	0.0206757
Insulin resistance	11	0.0114	0.0247826
Sphingolipid signaling pathway	11	0.0244	0.0441071
Osteoclast differentiation	11	0.027	0.0473684
Colorectal cancer	10	0.000538	0.00538
Long-term potentiation	10	0.000691	0.0054071
Chronic myeloid leukemia	10	0.00154	0.0083684
Estrogen signaling pathway	10	0.0128	0.0266667
Glucagon signaling pathway	10	0.0166	0.0320755
Acute myeloid leukemia	9	0.000938	0.0062533
Long-term depression	9	0.00154	0.0083684
RIG-I-like receptor signaling pathway	9	0.00332	0.0132308
Renal cell carcinoma	9	0.00332	0.0132308
Adipocytokine signaling pathway	9	0.0049	0.0158065

Apoptosis	9	0.0105	0.0238636
Endometrial cancer	8	0.00212	0.0096364
Glioma	8	0.00857	0.0217317
Central carbon metabolism in cancer	8	0.00938	0.0223333
Gastric acid secretion	8	0.0167	0.0320755
Circadian rhythm	7	0.000374	0.0046875
Sphingolipid metabolism	7	0.00541	0.0165588
Basal cell carcinoma	7	0.0114	0.0247826
mTOR signaling pathway	7	0.0195	0.0361111
Cytosolic DNA-sensing pathway	7	0.0247	0.0441071
Thyroid cancer	5	0.00891	0.0217317
Other types of O-glycan biosynthesis	5	0.0118	0.0251064
Glycosphingolipid biosynthesis - globo series	4	0.00377	0.013963
